# Supplementary material for: Stability of Flavonoid, Carotenoid, Soluble Sugar and Vitamin C in ‘Cara Cara’ Juice during Storage
Source: Foods. 2019 Sep 16;8(9):417. doi: 10.3390/foods8090417 (PMC6770534; doi:10.3390/foods8090417)
Supplement: Supplementary file 1 [file foods-08-00417-s001.pdf]

Table S1. Changes in the content of carotenoids (μg/mL) in "Cara Cara"juice during 16 weeks of storage at different temperature.

Week	mutatoxanthin	zeaxanthin	β-cryptoxanthin	ζ-carotene	β-carotene	13- or 15-cis -lycopene	lycopene	phytoene	phytofluene	cis-phytofluene	ester group 1	ester group 2	ester group 3	total carotenoid
4℃														
0	0.25±0.08a	0.59±0.07a	1.20±0.14a	0.72±0.08a	10.48±0.68a	0.35±0.04b	5.21±0.54a	215.69±5.21a	37.15±2.26a	24.60±1.52a	7.00±0.39a	5.10±0.22a	0.72±0.05a	309.06±11.28a
2	0.25±0.06a	0.48±0.06ab	1.00±0.08ab	0.58±0.06ab	9.73±0.47ab	0.36±0.03b	5.04±0.27a	209.55±6.78a	32.00±3.14a	27.66±1.88a	5.98±0.50ab	2.34±0.19b	0.64±0.05a	295.61±13.57a
4	0.24±0.03a	0.45±0.05ab	1.03±0.12ab	0.5±0.04b	9.21±0.80ab	0.35±0.03b	4.94±0.39a	205.14±7.38a	30.71±2.91a	27.75±1.05a	5.6±0.62abc	1.65±0.11c	0.60±0.04a	288.17±13.55a
6	0.24±0.04a	0.39±0.04b	0.83±0.06ab	0.48±0.03b	8.75±0.77ab	0.36±0.04b	4.74±0.54a	208.40±8.47a	28.18±3.21a	28.04±1.26a	4.85±0.33bc	0.99±0.08d	0.68±0.06a	286.93±14.93a
8	0.25±0.04a	0.39±0.05b	0.90±0.08ab	0.45±0.06b	8.67±0.69ab	0.52±0.05a	4.92±0.47a	201.89±10.22a	28.43±2.55a	27.65±1.87a	4.76±0.43bc	0.73±0.06de	0.59±0.05a	280.15±16.62a
12	0.22±0.05a	0.34±0.04b	0.90±0.07ab	0.52±0.03b	7.78±0.45b	0.52±0.04a	4.77±0.56a	196.08±9.88a	32.87±1.85a	24.07±1.47a	4.68±0.52bc	0.52±0.05e	0.74±0.06a	274.01±15.07a
16	0.22±0.04a	0.37±0.02b	0.92±0.05b	0.59±0.05ab	8.20±0.58ab	0.65±0.03a	4.65±0.52a	194.77±10.12a	28.18±1.22a	26.88±2.01a	4.00±0.35c	0.47±0.06e	0.76±0.05a	270.66±15.10a
20℃														
0	0.25±0.08a	0.59±0.07a	1.20±0.14a	0.72±0.08a	10.48±0.68a	0.35±0.04c	5.21±0.54a	215.69±5.21a	37.15±2.26a	24.6±1.52a	7.00±0.39a	5.10±0.22a	0.72±0.05a	309.06±11.28a
2	0.25±0.06a	0.43±0.02a	1.02±0.07ab	0.52±0.03bc	9.89±1.02a	0.35±0.03c	5.18±0.66a	208.24±6.79a	32.35±1.88a	27.43±2.55a	5.96±0.22ab	2.23±0.35b	0.52±0.04b	294.37±13.72a
4	0.27±0.05a	0.47±0.08a	1.04±0.06ab	0.55±0.05abc	8.97±0.78a	0.46±0.03c	5.09±0.61a	209.82±9.57a	31.11±2.47a	26.64±1.79a	5.50±0.34bc	0.96±0.15c	0.81±0.07a	291.69±16.05a
6	0.25±0.03a	0.43±0.05a	0.91±0.04b	0.38±0.02c	8.57±0.74a	0.68±0.05b	4.63±0.52a	206.82±8.99a	32.31±3.88a	26.74±2.06a	5.25±0.48bc	0.78±0.04cd	0.45±0.06b	288.20±16.92a

8	0.26±0.03a	0.41±0.05a	0.96±0.08ab	0.45±0.06bc	7.98±0.49a	0.67±0.05b	4.75±0.39a	202.57±8.79a	30.52±2.57a	26.23±2.11a	5.17±0.39bc	0.57±0.03cd	0.76±0.05a	281.30±15.09a
12	0.23±0.02a	0.44±0.02a	0.90±0.05b	0.46±0.04bc	8.37±0.55a	0.63±0.06b	4.88±0.61a	198.99±10.23a	28.65±2.44a	25.91±1.85a	4.42±0.27c	0.36±0.02d	0.89±0.02b	275.13±16.18a
16	0.26±0.04a	0.41±0.03a	0.91±0.03b	0.63±0.05ab	8.1±0.64a	0.85±0.04a	4.66±0.47a	197.52±7.59a	28.55±1.89a	24.87±2.03a	4.33±0.31c	0.35±0.02d	0.90±0.07b	272.34±13.21a
							30℃							
0	0.25±0.08a	0.59±0.07a	1.20±0.14a	0.72±0.08ab	10.48±0.68a	0.35±0.04c	5.21±0.54a	215.69±5.21a	37.15±2.26a	24.6±1.52b	7.00±0.39a	5.10±0.22a	0.72±0.05ab	309.06±11.28a
2	0.30±0.04a	0.51±0.05ab	1.09±0.08a	0.54±0.05bc	10.00±0.79a	0.41±0.02bc	5.12±0.62a	216.07±8.78a	31.55±3.25abc	27.76±1.57ab	5.70±0.66ab	1.98±0.14b	0.79±0.06ab	301.82±16.11a
4	0.28±0.03a	0.50±0.03ab	1.02±0.08a	0.57±0.04abc	9.29±0.86a	0.56±0.04ab	4.82±0.39a	210.43±9.58a	32.62±2.74bc	26.10±2.14b	5.34±0.35bc	0.79±0.08c	0.81±0.05a	293.13±16.41a
6	0.21±0.01a	0.48±0.06ab	1.09±0.09a	0.62±0.04abc	8.56±0.47a	0.57±0.04a	5.02±0.34a	202.76±8.36a	25.47±2.78bcd	31.58±1.88ab	4.63±0.28bcd	0.66±0.05cd	0.85±0.06a	282.50±14.46a
8	0.23±0.02a	0.43±0.05ab	0.98±0.06a	0.53±0.02c	8.72±0.84a	0.56±0.05ab	4.78±0.63a	197.65±9.25a	23.03±1.58cd	31.89±2.06ab	4.26±0.39cd	0.62±0.04cd	0.82±0.04a	274.50±15.03a
12	0.22±0.06a	0.42±0.06ab	0.98±0.04a	0.64±0.07abc	8.71±0.39a	0.63±0.07a	4.83±0.44a	198.56±6.99a	21.85±2.66d	34.90±2.98a	3.94±0.33cd	0.39±0.05d	0.61±0.07b	276.68±14.21a
16	0.20±0.04a	0.38±0.02b	0.96±0.05a	0.75±0.04a	8.36±0.48a	0.59±0.04a	4.63±0.50a	196.59±7.88a	20.16±1.97d	35.63±3.58a	3.80±0.25d	0.36±0.02d	0.81±0.04a	273.22±14.91a
							40℃							
0	0.25±0.08a	0.59±0.07a	1.2±0.14a	0.72±0.08a	10.48±0.68a	0.35±0.04c	5.21±0.54a	215.69±5.21a	37.15±2.26a	24.6±1.52a	7.00±0.39a	5.10±0.22a	0.72±0.05a	309.06±11.28a
2	0.24±0.03a	0.49±0.05ab	1.19±0.11a	0.70±0.04a	10.26±0.77a	0.52±0.04bc	5.18±0.55a	212.09±10.02a	30.62±3.01a	28.02±1.77ab	5.92±0.22b	1.25±0.11b	0.85±0.08a	297.33±16.80a
4	0.17±0.02a	0.45±0.05abc	1.06±0.09a	0.79±0.05a	9.97±0.57a	0.65±0.05ab	4.99±0.54a	210.79±10.35a	30.75±2.88a	27.16±3.06ab	5.75±0.36b	0.89±0.06c	0.91±0.06a	294.33±18.14a

6	0.20±0.02a	0.39±0.02bc	0.98±0.08a	0.77±0.05a	9.17±0.59a	0.64±0.05ab	4.72±0.67a	205.98±9.84a	29.68±3.01a	29.00±2.15ab	4.60±0.33c	0.66±0.05cd	0.80±0.05a	287.59±16.91a
8	0.18±0.04a	0.35±0.04bc	0.95±0.06a	0.84±0.07a	9.07±0.45a	0.76±0.05a	4.95±0.35a	208.55±7.94a	29.23±2.26ab	28.82±1.87ab	4.11±0.32cd	0.37±0.01de	0.85±0.04a	289.03±13.50a
12	0.20±0.02a	0.32±0.03c	0.93±0.05a	0.89±0.09a	9.21±0.54a	0.73±0.08a	4.89±0.44a	207.73±9.33a	28.68±3.55ab	31.58±3.02ab	3.49±0.26d	0.31±0.02e	0.75±0.06a	289.71±17.49a
16	0.21±0.02a	0.35±0.02bc	0.95±0.07a	0.84±0.05a	8.73±0.94a	0.65±0.05ab	4.98±0.37a	201.56±9.55a	19.84±2.05b	34.99±4.01b	3.48±0.19d	0.27±0.02e	0.72±0.05a	277.57±17.39a

Values are expressed as mean ± SD, n= 3. Values followed by different letters in the same line are significantly different ($p < 0.05$).

ester group 1, belonged to β-cryptoxanthin esters ,including peaks 13, 14, 16, 17, 20, accroding to Fig. 1.

ester group 2, belonged to epoxy carotenoids esters, including peaks 4, 5, 7, 8, 11, 12, 15, 18, 21, 22, accroding to Fig. 1.

ester group 3, the unknown ester, peak 10.

cis- phytofluene, including peaks 26, 27 and 29, accroding to Fig. 1.

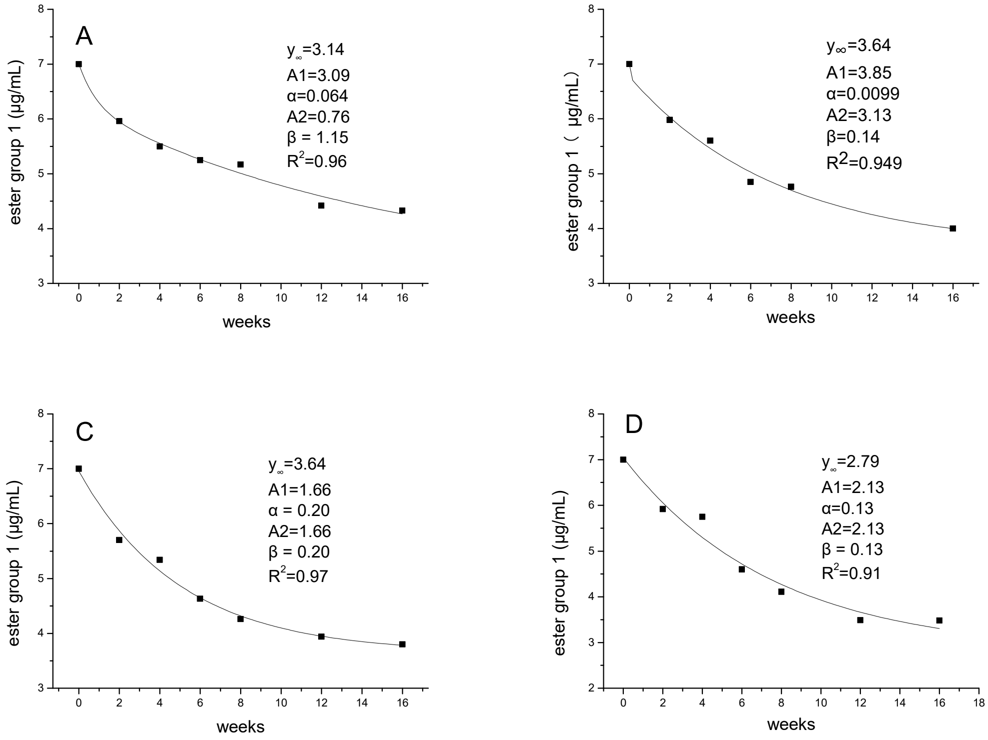

Figure S1. Biexponential fitting with eq 1 of the experimental data obtained for the degradation of ester group 1 in Cara Cara juice during 16 weeks storage at 4℃ (A), 20℃ (B), 30℃ (C) and 40℃ (D), respectively.

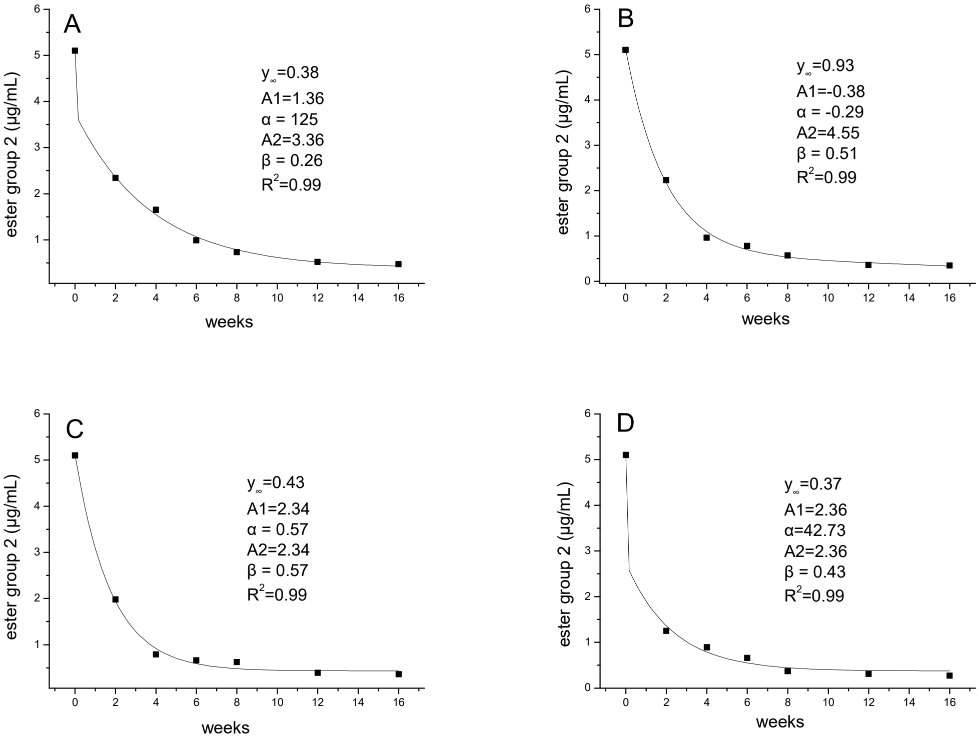

Figure S2. Biexponential fitting with eq 1 of the experimental data obtained for the degradation of ester group 2 in Cara Cara juice during 16 weeks storage at 4℃ (A), 20℃ (B), 30℃ (C) and 40℃ (D), respectively.
